# Rarity Value and Species Extinction: The Anthropogenic Allee Effect

**DOI:** 10.1371/journal.pbio.0040415

**Published:** 2006-11-28

**Authors:** Franck Courchamp, Elena Angulo, Philippe Rivalan, Richard J Hall, Laetitia Signoret, Leigh Bull, Yves Meinard

**Affiliations:** Ecologie, Systématique, et Evolution, UMR CNRS 8079, Universite Paris-Sud, Orsay, France; Institute of Zoology, United Kingdom

## Abstract

Standard economic theory predicts that exploitation alone is unlikely to result in species extinction because of the escalating costs of finding the last individuals of a declining species. We argue that the human predisposition to place exaggerated value on rarity fuels disproportionate exploitation of rare species, rendering them even rarer and thus more desirable, ultimately leading them into an extinction vortex. Here we present a simple mathematical model and various empirical examples to show how the value attributed to rarity in some human activities could precipitate the extinction of rare species—a concept that we term the anthropogenic Allee effect. The alarming finding that human perception of rarity can precipitate species extinction has serious implications for the conservation of species that are rare or that may become so, be they charismatic and emblematic or simply likely to become fashionable for certain activities.

## Abstract 2

A model shows how the value that humans place on rarity fuels disproportionate exploitation of rare species, rendering them even rarer and thus more desirable, ultimately leading them into a vortex of extinction.

## Introduction

Overexploitation of living species (i.e., human exploitation exceeding the species' regeneration capacity) is a major threat to biodiversity [1], yet theory predicts that economic extinction (exploitation cessation) will usually precede ecological extinction (population disappearance). As populations become more sparse, it is increasingly costly to exploit them, and exploitation ceases to be beneficial [2]. In the absence of natural extinction risks at low population size (e.g., demographic stochasticity), exploitation cessation allows for the species' recovery. However, less-abundant species could suffer disproportionately from exploitation if their rarity makes them systematically more valuable. We postulate that because rarity makes living species attractive, their (over)exploitation can remain profitable, rendering such species even rarer, and driving them to extinction.

This human-generated feedback loop is very similar to the Allee effect [3,4], an important process in basic ecology and applied conservation biology. Whereas ecologists have historically focused on negative density dependence, they are increasingly realising that an ever-growing number of species suffer from positive density dependence at low population density. In many animal and plant species, individual reproduction and survival is diminished in small populations through various mechanisms including mate shortage, failure to optimize the environment, or lack of conspecific cooperation. Populations suffering from Allee effects may exhibit negative growth rates at low densities, which drives them to even lower densities and ultimately to extinction. A typical example is that of obligate cooperative breeding species, which need group members to enable them to raise offspring, survive predators, and/or forage cooperatively, and fail to do so efficiently when their numbers drop [5].

Although studies on Allee effects are continuing, it has been generally accepted that the Allee effect is intrinsic to the species concerned, which express it naturally at low density. Therefore, human activities cannot create an Allee effect; at most, they can push species into density ranges where their natural Allee effect will be expressed. On the contrary, we show here that humans can induce a purely artificial Allee effect in rare species through the “paradox of value” [6]. We call it the anthropogenic Allee effect (AAE). Although familiar to economists, the paradox of value—also called the “water and diamonds paradox” (water has much value in use but none in exchange, while the opposite is true for diamonds)—is absent from ecological theory. Here we will provide a mathematical model to demonstrate how an AAE can, in theory, emerge in wildlife-related trade as soon as rarity acquires value. We then identify a number of human activities where an AAE can occur and use examples to illustrate each of them.

## Model and Results

### Theoretical Framework

The AAE is founded on two fundamental assumptions: (i) there is a positive correlation between species rarity and its value, and (ii) this correlation fuels sufficient demand to ensure that the market price exceeds the escalating costs of finding and harvesting a declining species. If these simple conditions are met, harvesting reduces the population of the rare species, increasing its rarity and therefore its value, which stimulates further harvesting and drives the species into an extinction vortex.

**Figure 1:**
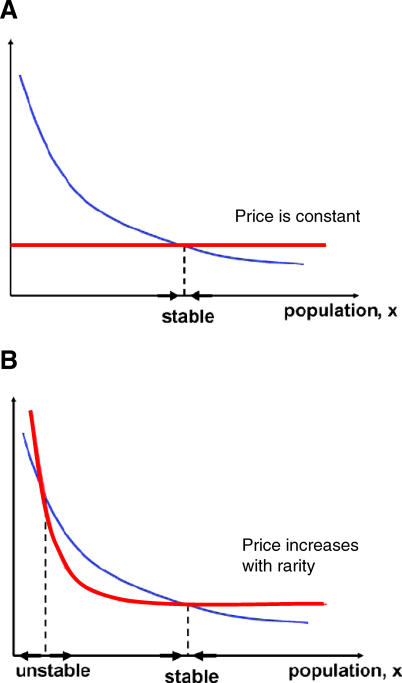
Demonstration of an Anthropogenic Allee Effect with a Simple Model of an Exploited Population The price (red, thick line) and cost (blue, thin line) per unit harvest in unit time as a function of the population density *x* when (A) the price is independent of *x* and (B) the price increases with rarity. The system is in equilibrium whenever the red and blue lines meet, and the bold arrows represent how the population responds when perturbed from equilibrium. In (B), an increased price at low population density induces an Allee Effect.

We added these assumptions as a simple modification to the Gordon-Schaefer model [7] of resource exploitation to assess their effect on population density equilibrium. The model applies to populations subject to open-access (i.e., unregulated) exploitation [2], and in particular, to the dynamics of rare species subject to poaching [8]. In the absence of harvesting, the population *x* grows according to the logistic equation with rate *r* and carrying capacity *K*. It is harvested at a rate proportional to both the population level *x* and the hunting effort *E*. The rate of change of hunting effort is assumed to be proportional to economic rent or profit, which is the difference between the price obtained for the harvest and the total cost of hunting. This can be expressed mathematically as

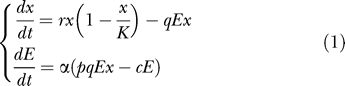
where *q* is the catchability of the species, α is a measure of how rapidly hunters respond to changes in profit, *p* is the (constant) price obtained per unit harvest, and *c* is the cost per unit effort. Provided that one would make a profit when hunting the population at carrying capacity (i.e., *pqK* > *c*), this system has a globally stable equilibrium. The population is never hunted to extinction, because cost per unit harvest in unit time, *c*/*qx*, becomes very large as *x* tends to zero (Figure 1A).

If, however, the price of a unit harvest is an increasing function of the species rarity (i.e., *p* = *p*(*x*)*, dp*/*dx* < 0)—or more precisely, if below some critical population density, someone is always willing to pay enough to offset the costs of finding and hunting the species *(p(x)* > *c*/*qx* for small *x)*—then an unstable equilibrium exists (Figure 1B). Below this equilibrium population density, hunting further reduces the population, which increases the price of a unit harvest, which in turn increases the hunting effort, and the species is driven into an extinction vortex.

These results can also be understood within the framework of supply and demand theory. Clark [2] showed that the open-access exploitation model can be solved at equilibrium to obtain a relation between yield *(qEx)* and price, the equilibrium supply curve. That supply curve for an exploited population is backward-bending, corresponding to overexploitation when the species is hunted at levels above the maximum sustainable yield. In traditional models, the demand curve is usually elastic (i.e., the quantity purchased is sensitive to changes in price), and hence only intersects the supply curve once. It is this price that determines the equilibrium population size and exploitation effort. However, our assumption that people are willing to pay more for rare species results in a demand curve which becomes increasingly inelastic with rarity, and the supply curve is cut twice, exactly as in Figure 1B: the population is harvested to extinction if its drops below the lower equilibrium.

### Empirical Examples

**Figure 2:**
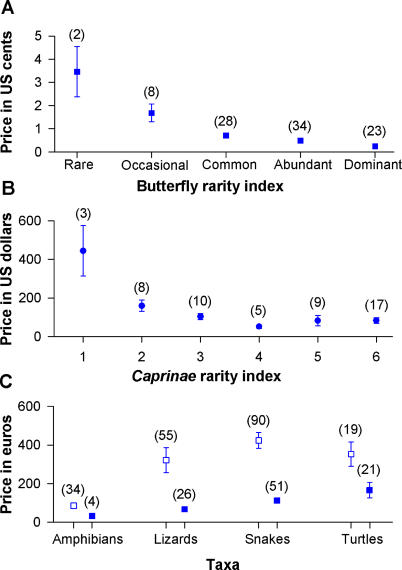
Empirical Examples of Activities where the Price of Species Is Related to Rarity or Perceived Rarity (A) Prices of collectible butterflies in Papua New Guinea (modified from [9]). (B) Hunting trophy prices of 57 *Caprinae* taxa. (C) Selling prices of exotic pet species according to CITES status. Species that have a CITES status (open squares) are more expensive than species with no CITES status (solid squares). Prices were standardized by dividing by (A) male wingspan, (B) trophy size, and (C) adult weight (see text for details). Vertical bars: standard error; sample size in parentheses.

Data showing a positive relationship between price and rarity are scarce but do exist for a number of nature-related economic activities (we present some analyses in Figure 2). The second assumption of the AAE, that prices increase with rarity faster than the exploitation costs, may be more difficult to test. In general, one might expect that increasing exploitation costs lead to increasing prices, which in turn results in a drop in demand. However, other factors act to reinforce the demand, for example, when it becomes fashionable to acquire a rare item (see examples below). The second assumption is therefore fulfilled if there are always a few consumers willing to acquire the last individuals at any price. In this case, ecological extinction (the end of the species) will precede economic extinction (cessation of exploitation), contrary to the predictions of classical theory (the simple Gordon-Schaefer model [7]).

We claim that a number of human activities can create an AAE. Below, we develop some examples that illustrate under what conditions people are willing to pay (or risk) substantial amounts for the satisfaction and/or prestige of acquiring rare species. It is, however, important to stress that correlation does not necessarily prove causation. The only true test of the effect would be to track changes in a particular species' demand curve with rarity, which is not feasible in a dynamic system.

#### Collections.

The most straightforward example of a nature-related activity where rarity is valued is that of hobby collections, where the rarest items are the most valued and thus demand the highest prices. As the value of a rare item increases, more time, effort, or resources may be devoted to trying to acquire it, increasing the pressure on the species as it becomes rarer. The collection of butterflies in Papua New Guinea illustrates this point [9], whereby the price of butterflies sold by villagers to insect collectors were correlated with rarity, even when corrected by size (price divided by male wing span; Kendall concordance test for nonparametric data τ = −0.39, *p* < 0.0001) (Figure 2A). Therefore, it is the rarity of the butterflies, not their size, that drives the price of these collection items. Collectors of wildlife items such as other insects, bird eggs, mollusk shells, or orchids often adhere to this rule. For example, the collection of bird eggs threatens many rare species in the United Kingdom, and this practice continues despite the threat of financial penalties and/or jail terms [10]. When species are protected by local laws and/or by international trade treaties, the high price that the rarest species can fetch on the collection market is a powerful incentive to poachers and smugglers to seek and illegally sell the most expensive (rare) species, constituting a real threat to some of these species.

Scientists have historically been, and in some cases still are, among these enthusiastic collectors of natural specimens. Following the overexploitation of the great auk Pinguinus impennis for food and feathers, the species became very rare. As a consequence, these birds became a valuable item for collectors—among them, ornithologists and museum administrators, who were eager to acquire eggs or skins of the rare and soon to be extinct bird, thereby precipitating its extinction [11]. The great auk provides a possible example of an AAE leading to a species extinction and should serve as a warning for currently threatened species.

#### Trophy hunting.

Trophy hunting represents another form of collection. For thousands of years, several cultures have valued trophies as a sign of manhood and virility. Species that were difficult to kill symbolized power, because power was required to kill them. However, because sophisticated firearms are now used, the emphasis of hunting has shifted from dangerous to rare animals. Rarer species are harder to find, so greater hunting skill—and greater wealth—is required, and greater prestige is gained by killing them. We compared the standardised quality of trophies of 57 species and subspecies of *Caprinae* hunted for their trophies with the average price of the trophy hunting in the 2006 season, as proposed by various hunting tour operators on the Internet. The prices of the hunting trophies were standardised by dividing the average price for each taxon by the current Safari Club International (SCI) world record for these taxa. SCI is a standardised measurement method for trophies developed by trophy-hunting societies and is designed to allow interspecific comparisons of trophy quality. Thus, the price of a trophy should depend solely on its SCI value. We then compared this standardised price for species according to their perceived rarity through an index of rarity constructed by attributing points to each species according to their protection status. We accounted for the World Conservation Union (IUCN: critically endangered = 0 point; endangered = 1; vulnerable = 2; others = 3), the Convention on International Trade in Endangered Species of Wild Fauna and Flora (CITES: Appendix I = 0; Appendix II = 1; Appendix III = 2; no Appendix = 3), and local protection (yes = 0; no = 1). Points were added, and the cumulative score was used as an indication of the perceived rarity of the taxon; the lower the score, the rarer the taxon. Once again, our results show that hunting trophy prices are correlated with rarity, regardless of size (Kendall concordance test for nonparametric data τ = −0.34, *p* < 0.0001): the rarer the trophy, the more valuable and expensive it is (Figure 2B). For wild sheep alone, wealthy hunters are willing to pay more than US$400,000 at auctions to shoot a rare animal [12], because few of their peers will be able to do so, and they will gain social prestige in being one of the few who can afford it. Very few hunting permits are delivered for protected species, making the animals even more attractive to trophy collectors and possibly stimulating illegal hunting.

#### Luxury items.

**Figure 3:**
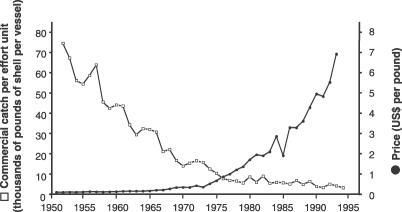
California Commercial White Abalone Haliotis sorenseni Landings for 1972–1992 The price exponentially increased as catch decreased. The catch takes the fishing effort into account, so that decreased catch does not come from a diminishing effort. Results are given as weight in shell divided by the number of ships (open squares) and price (filled circles) [27].

The consumption of rare species as luxury food items is another way of displaying wealth and/or social status. The rarer the item, the more expensive it is, and the more prestige is gained by its acquisition. When closing deals, wealthy Asian businessmen wishing to display their affluence will pay large amounts of money to eat a plate of lips of a large Napoleon wrasse, Cheilinus undulatus (a single pair of lips costs US$250). By the mid 1990s, Napoleon wrasse became the most sought-after reef fish in the world [13], and is currently number one on the “top ten most-wanted species” list published by the World Wide Fund for Nature. Populations in South East Asia are now extinct on many reefs, and very few large individuals survive in the remaining fragmented populations [13]. The caviar obtained from different sturgeon species provides another example for the potential for the feedback loop we describe here. Not only are all sturgeon species currently on the IUCN Red List and CITES Appendices [14,15], but the price of the caviar is correlated with its rarity [16]. Abalones, of which six species suffer from overfishing on the Pacific coast of North America, are another illustration. Considered a delicacy in California, white abalones, the rarest of the six abalone species, have declined by over 99.99% due to increasing overfishing, in part illegal (the fishery was closed in 1996), while at the same time, prices have escalated [17–19]. Even taking fishing effort into account, the volume of abalones fished is inversely proportional to the price (power regression model, *F*_40,2_ = 139.96; *p* < 0.0001) (Figure 3). Although white abalones were the first marine invertebrate on the United States endangered species list in 2001, this species could become extinct within a decade unless extraordinary recovery measures are implemented [18]. Due to the demand for other types of luxury items, such as exotic woods, furs, turtle shells, or snake and crocodile skins, many other species are likely to be vulnerable to AAEs in this context.

#### Exotic pets.

Another activity that can lead to an AAE is exotic pet ownership, which is an increasingly important part of the wildlife trade business. Reptile, bird, monkey, and felid pets are becoming ever more fashionable in some parts of the world, with the rarest species being especially sought after. Given that high levels of mortality occur during the capture or transfer of traded species due to inadequacies in care, the massive volumes of live species that are traded are likely to exert considerable pressure on the target populations. Unlike the trophy hunting market, the exotic pet market involves many taxa, including arachnids, molluscs, insects, fish, and other vertebrates. For example, aquarium “hardcore” collectors seek rare items such as the peppermint angelfish *Centropyge boylei,* which sell for over US$10,000. Even though the trade of many such animals is illegal, smugglers generally face low penalties and therefore continue to deplete endangered populations for large amounts of money.

A recent article reports that immediately after being described in the scientific literature, the turtle Chelodina mccordi from the small Indonesian island of Roti and the gecko Goniurosaurus luii from southeastern China became recognized as rarities in the international pet trade, and prices in importing countries soared to highs of US$1,500 to US$2,000 each [20]. They became so heavily hunted that today, C. mccordi is nearly extinct in the wild and G. luii is extirpated from its type locality [20].

Some individuals in the wildlife trade business believe that the declaration of a species as endangered by a conservation organization provides official proof that the species is rare and therefore more valuable. Hence, paradoxically, declaring a species endangered may make it more desirable and thereby increase the likelihood of exploitation. We compared the selling prices of exotic amphibian and reptile species sold as pets in early 2006 by the largest herpetologist retailer in France (which sells to the other retailers in Europe as well as to the public) according to the CITES status of the species (Figure 2C). When corrected by adult weight, species that have a CITES status were found to be significantly more expensive (analysis of variance [ANOVA]; amphibians *p* = 0.0024; lizards *p* < 0.0001; snakes *p* < 0.0001; and turtles *p* < 0.0001) than species with no CITES status, probably as a consequence of them being considered more valuable as a result of their rarity.

**Figure 4:**
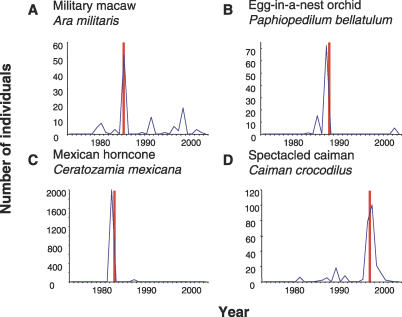
Illustration of the Increased Demand Associated with Perceived Rarity Volumes of illegal trade of four species examples [a bird (A), two plants (B and C), and a reptile (D)], which have undergone a change in CITES status. The year that the change in status took effect is indicated by the red line.

To further investigate the effect of CITES status on perceived value of the species, we analyzed the CITES database to assess the effect on illegal trade of a change of status, for species passing from Appendix 2 (species whose survival might be compromised if trade was not restricted) to Appendix 1 (very restricted trade, species threatened with extinction, perceived as the rarest). Of the 133 plant and animal species that have undergone this change over the past 30 years, 44 have never been reported as being traded illegally. Interestingly, classification of some species as highly endangered resulted in an increase in their illegal trade (Figure 4): of the 89 remaining species, 23 (25.8%) have shown a marked peak of illegal trade during the period (±1 y) corresponding to the change in CITES status (because the change is officially proposed 9–15 months before the application, poachers can be informed one full year in advance). This is a compelling illustration of both the increased attractiveness of rarer species and the exacerbated threat this classification may have on species becoming rare if they cannot be properly protected.

#### Ecotourism.

Ecotourism ventures have expanded greatly in recent years, with the public increasingly wanting to experience a closeness to natural ecosystems or species. Such activities often involve encountering and/or observing rare species. Given that some ecotourism activities have been shown to generate disturbances that are detrimental to the fitness of observed species [21–23], we can assume that rare species, especially those that are charismatic, will be disproportionately impacted upon by ecotourism. Consequently, activities such as observing rare birds, whales, primates, or nesting sea turtles have the potential to generate an AAE, especially when the animals are globally rare but with reliable sightings locally. For example, Bain [21] studied the relationships between the number of killer whales Orcinus orca in the Southern resident population (eastern North Pacific) and the number of boats registered for conducting killer whale watching tours. He found a significant inverse relationship between the number of boats observed in one year with the whale population size recorded the subsequent year. Motorized boats are known to cause disturbances to whales and lower their fitness [21]. More interestingly, there was also an inverse relationship between the decreasing whale population size recorded during one year, and the increasing size of the boat fleet the next year, indicating that contrary to expected economics, the increasing rarity of that population of killer whales did not immediately stop whale watching but may have in fact stimulated it [21]. In 2001, the number of boats in the commercial whale watching fleet exceeded the number of killer whales in the population.

The Capercaillie (Tetrao urogallus) is a large gamebird that inhabits Scottish forests, where its population has dropped precipitously from 20,000 to 900 birds in the past 30 years [24]. Mating takes place just a few times each spring at the display grounds, or leks, of the males. Given the rarity of these birds, there is great interest in observing these leks amongst British birdwatchers, and disturbance of leks is thought to be a serious threat to the survival of the Scottish population.

#### Traditional medicine.

Traditional medicine uses many rare and endangered species. Although other aspects may influence ingredient choice, rarity certainly plays a role [25] and may therefore result in an AAE. In western Japan, the red morph of Geranium thunbergii, a flower widely used for treating stomach problems and diarrhea, is common, whereas the white morph is rare. The morph frequency is the opposite in eastern Japan, with the white morph being common and the red morph rare. People in western Japan believe that the medicinal efficiency of the “rare” white morph is better, whereas those in eastern Japan consider the “rare” red morph superior (Tetsukazu Yahara, personal communication). This geographic difference in people's beliefs is likely to exert strong selective pressure on flower colour and offers a good illustration of the preference for rarity and its perceived medicinal virtues.

The Chinese bahaba *Bahaba taipingensis* provides another example of the effect that exploitation for traditional medicine can have on rare populations. Used for the prevention of miscarriages, the swimbladder of this fish is highly valued in Asia; as indicated by the name “soft gold”, which was assigned to it by fishers as it became increasingly rare over the last four decades [25]. As the exploitation of this fish intensified, its increasing rarity made its value escalate to such a level that despite less than half a dozen fish being caught per year in the 1990s (less than 1% of the amount caught in the 1960s), 100–200 boats continued to target this fish [25]. One large swimbladder was sold in the 1980s for US$64,000 [25]. In 2001, some 70 years after it was first reported in the scientific literature, this species was virtually extinct [26]. At that time, the occasional fish that was caught every few years yielded a swimbladder with a value that on the top retail market, weight for weight, exceeded that of gold by seven times [25].

## Conclusion

We have identified six different types of human activities that have the potential to induce an AAE, but there are likely to be others. It is important to realize that an AAE has the potential to target not only the most charismatic and emblematic species, but also the most inconspicuous invertebrate, as long as rarity renders it fashionable to exploit for one reason or another. Furthermore, species that are currently not of concern could very well be in the near future.

Because among the activities presented here, several are primarily stimulated by people interested in nature, it is important that these people are aware of and have an understanding of the potential effect their actions may have on the very species they appreciate. Consequently, informing potential ecotourists, collectors, and pet owners may in part facilitate the process of reducing the likelihood of an AAE and thus the impact on the species that are the targets of these activities. However, activities that relate to prestige or tradition may require more dramatic actions—including strengthened regulations and targeted, adapted information—to decrease the likelihood of AAEs in the target species.

How the trade of rare species should be regulated is a vast and ongoing debate. The finding that rarity itself could be a criterion for immediate threat to a species because of the psychological and economic value people attach to it is, however, a new and important piece of information in the battle to preserve biodiversity. At the very least, this finding should lead to the realization that declaring a species too rare to be subjected to legal transactions could be dangerous for the species if it cannot be fully protected. At most, it is hoped that such information could change our rationale on the manner in which biodiversity is perceived and exploited.

## Abbreviations

AAE: anthropogenic Allee effect
